# A Novel Bi-Functional Fibrinolytic Enzyme with Anticoagulant and Thrombolytic Activities from a Marine-Derived Fungus *Aspergillus versicolor* ZLH-1

**DOI:** 10.3390/md20060356

**Published:** 2022-05-27

**Authors:** Lihong Zhao, Xiuping Lin, Jingyun Fu, Jun Zhang, Wei Tang, Zengguo He

**Affiliations:** 1 School of Medicine and Pharmacy, Ocean University of China, Qingdao 266003, China; zhaolihong1990@163.com (L.Z.); fujingyun@ouc.edu.cn (J.F.); zhangjun841013@aliyun.com (J.Z.); tangwei@ouc.edu.cn (W.T.); 2 CAS Key Laboratory of Tropical Marine Bio-Resources and Ecology, Guangdong Key Laboratory of Marine Materia Medica, South China Sea Institute of Oceanology, Chinese Academy of Sciences, Guangzhou 510301, China; xiupinglin@scsio.ac.cn; 3 Marine Biomedical Research Institute of Qingdao, Qingdao 266071, China; 4 Qingdao Bioantai Biotechnology Co., Ltd., Qingdao 266000, China

**Keywords:** *A. versicolor* ZLH-1, fibrinolytic enzyme, anticoagulant activity, thrombolytic activity

## Abstract

Fibrinolytic enzymes are important components in the treatment of thrombosis-associated disorders. A new bi-functional fibrinolytic enzyme, versiase, was identified from a marine-derived fungus *Aspergillus versicolor* ZLH-1. The enzyme was isolated from the fungal culture through precipitation with ammonium sulfate at 90% saturation. Additionally, it was further purified by DEAE-based ion-exchange chromatography, with a recovery of 20.4%. The fibrinolytic enzyme presented as one band on both SDS-PAGE and fibrin-zymogram, with a molecular mass of 37.3 kDa. It was elucidated as a member of metalloprotease in M35 family by proteomic approaches. The homology-modeling analysis revealed that versiase shares significant structural homology wuth the zinc metalloendopeptidase. The enzyme displayed maximum activity at 40 °C and pH 5.0. The activity of versiase was strongly inhibited by the metalloprotease inhibitors EDTA and BGTA. Furthermore, versiase hydrolyzed fibrin directly and indirectly via the activation of plasminogen, and it was able to hydrolyze the three chains (α, β, γ) of fibrin(ogen). Additionally, versiase demonstrated promising thrombolytic and anticoagulant activities, without many side-effects noticed. In conclusion, versiase appears to be a potent fibrinolytic enzyme deserving further investigation.

## 1. Introduction

Thrombosis has long been an acute case among the cardiovascular diseases (CVDs) [1]. Fibrin, the major protein component of blood clots, is formed from fibrinogen via proteolysis by thrombin [2]. Fibrinogen may cause thrombus independently once its level is above the normal range [3]. Intravascularly, thrombosis is associated with the imbalance between the two processes, coagulation and fibrinolysis. Under some disordered physiological conditions, the balance is broken, then clots occur, and thrombosis takes place eventually.

For treating formed blood clots, surgical intervention has been accepted as an effective therapeutic strategy because of its effectiveness in recanalization. However, it is either expensive or burdensome in hospitalization that necessitates the provision of specialist staff and catheterization [4,5]. In contrast, intravenous pharmacological or oral administration for thrombolysis treatments is minimally invasive or non-invasive, less expensive, simpler than intervention, and theoretically can be conducted immediately, even in an ambulance. Different from anticoagulants and antiplatelets, thrombolytic enzymes target at pre-existing thrombus with high degradation rate [6]. Accordingly, the interests on thrombolytic enzymes have been largely evoked thanks to their established clinical records in antithrombolytic application.

Based on the mechanisms of action, thrombolytic enzymes are classified into two groups. One group includes plasminogen activators (PAs), which activate plasminogen into active plasmin to degrade fibrin (indirect action). Another group covers the plasmin-like fibrinolytic enzymes that directly degrade fibrin [7]. Although PAs are still used in thrombolytic therapy today, they have expensive prices and undesirable side-effects, such as largely being discouraged by PAI-1 (plasminogen activator inhibitor-1) [7], bleeding tendency, short half-life, low specificity to fibrin, perfusion delay, etc. [8]. Unlike the popularly used PAs, the fibrinolytic enzymes are scarcely used in clinics. It is noted that some progress has been made on fibrinolytic enzymes, and even preclinical trials have been encouraged in a few cases [9]. Nevertheless, the ultimate fibrinolytic enzymes, ideally facilitated with both reliable clot-busting capacity and decent safety profiles, are still beyond reach thus far [6]. Therefore, continuous efforts are needed in hunting new resources for efficacious, cost-effective, yet safe thrombolytic drugs. Since for years, a panel of potent fibrin(ogen)olytic enzymes have been well documented or established in bacteria and fungi [10], the exploration of new fibrinolytic by microbes in the untouched niches may shed light on this need.

In a recent search for fibrinolytic enzymes by screening marine fungi, an isolate ZLH-1 was obtained from sponge *Callyspongia* sp. sample collected in the South China Sea. The strain was identified as *Aspergillus versicolor*. Its fibrinolytic enzyme versiase was documented herein, together with the approaches on the isolation, purification, and characterization of the enzyme, as well as with its potentials in anticoagulant and thrombolytic applications. The production of fibrinolytic protease by *A. versicolor* has not been reported yet.

## 2. Results and Discussion

### 2.1. Enzyme Purification, SDS-PAGE, and Zymography

**Figure 1:**
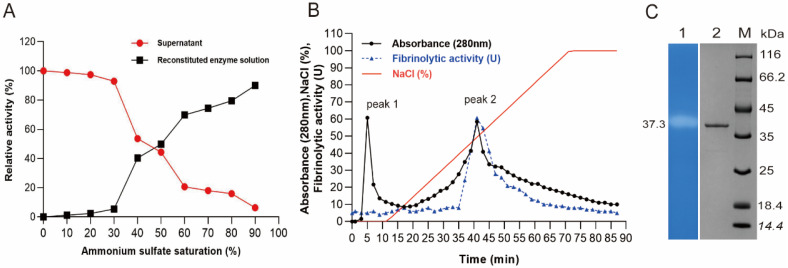
Purification of the fibrinolytic enzyme (versiase) from *A. versicolor* ZLH-1 culture supernatant. (**A**) The purification of versiase from supernatant through ammonium sulfate precipitation; (**B**) anionic exchange chromatography of versiase by DEAE-80S (phenyl) column; (**C**) SDS-PAGE analysis of purified enzyme and fibrin-zymogram. Lanes: M—protein marker; 1—fibrin-zymogram of versiase; 2—versiase.

A new fibrinolytic enzyme, versiase, was isolated and purified from the supernatant of the fermentation broth simply by a two-step purification procedure as described. The relationship between the relative enzymatic activity of AS precipitation and the saturation of AS was shown in Figure 1A. Obviously, enzyme activity in the precipitation is positively related to the saturation degree of ammonium sulfate (AS), and it approached 90% when the saturation of AS was at 90% at 4 °C. This saturation of AS was applied to isolate versiase in the fermentate supernatant of *A. versicolor* ZLH-1 thereafter. The results of SDS-PAGE showed that there were merely a few proteins bands existing in the fermentation broth (Figure S1A,B), indicating that the protein profile of the fermentate of ZLH-1 was quite simple.

The crude enzyme preparation was subjected to DEAE-80S (phenyl) ion-exchange chromatography with several proteins detected in the eluted fractions accordingly, one of which exhibited fibrinolytic activity (Figure 1B). The results showed that versiase was eluted into the fractions from 34 to 46, since the UV absorption and the enzymatic activity overlapped consistently in these fractions. The pooled fractions with enzymatic activity were further analyzed by SDS-PAGE, and merely a single band identified in the pooled fractions corresponded to peak 2 (Figure S1C), which verified that the ion-exchange chromatography purified versiase effectively by enriching it into peak 2. Through the entire purification process, versiase was purified 18.3-fold, with 1.16 mg enzyme obtained and a yield of 20.4% achieved ultimately derived from 500 mL of culture medium (Table 1), which is higher than that of other fibrinolytic proteases by the similar method as reported [11,12]. Interestingly, under the given conditions of fermentation, broth of *A. versicolor* ZLH-1 presented such a simple protein profile that the isolation and purification of versiase were eased by a mere combination of AS precipitation and DEAE-80S (phenyl) ion-exchange chromatography, even without involving of any other routine approaches such as gel filtration [12] and hydrophobic interaction chromatography [13], etc. Considering the simplicity of upstream-submerged production and the cost-effective manipulation for downstream manipulation, *A. versicolor* ZLH-1 is doubtlessly a potent producer for future explorations regarding the expression, as well as the engineering for the fibrinolytic enzymes of interest.

The homogeneity of the purified enzyme was determined by SDS-PAGE, which exhibited one single band with an apparent molecular mass around 37.3 kDa (Figure 1C). Parallelly, in fibrin zymography of the identical purified enzyme, a clear digested region was observed at the same position as shown in SDS PAGE (Figure 1C). This result clarified that it is the purified versiase that presents the fibrinolytic enzymatic activity.

### 2.2. The Identification and Modeling of Versiase

**Figure 2:**
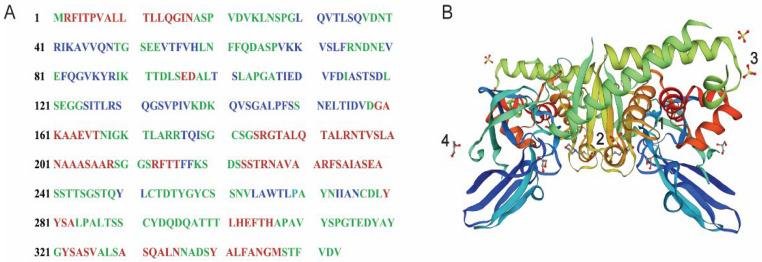
Predicted structure of versiase derived from CDS sequencing. (**A**) The amino acid sequence and secondary structure. Red indicates alpha helix, blue indicates beta-sheet, and green indicates random coil, respectively; (**B**) the model presents in a 3D structure representation. 1. 2 × Zn^2+^: (Zinc ion, non-covalent), 2. 2 × MES(2-(N-moroholino)-ethanesulfonic acid, non-covalent), 3. 6 × SO_4_^2-^ (sulfate ion, non-functional Binders), 4. 4 × GOL (glycerol, non-functional Binders).

The versiase band was excised from the gel (Figure 1C) and subjected to proteomic analysis using LC-ESI-MS/MS. Among the reference proteins listed, versiase was close to the DN6665 (Band 6, Supplementary Table S1), which has a 353 aa sequence (Figure 2A) and shares high similarity to the sequence of metalloprotease (M35 family) from *A. versicolor* (CBS 583.65). Its possible secondary structure was assessed using PredictProtein, and was composed of 29.75% alpha helix, 21.81% beta-sheet, and 48.44% random coil (Figure 2A). A homology modeling approach for constructing the 3D protein structure was employed. The model of 2.0 Å zinc metalloendopeptidase from *Aeromonas salmonicida* (SMTL ID: 2 × 3a.1) was evaluated as a reliable model by the GMQE (Global Model Quality Estimate) and QMEAN (Qualitative Model Energy Analysis) was analyzed as 0.55 and 0.60 ± 0.05 [14]. The predicted protein 3D structure was illustrated in Figure 2B.

Interestingly, as aforementioned, versiase falls into metalloproteases in the M35 family, which distinguishes itself from the serine-proteases nature found in plasminogen activators, as well as in the most of the direct fibrinolytic enzymes [8]. Doubtlessly, the non-serine-proteases nature of versiase deserves further investigation, both mechanism wise and thrombolytic orientated.

### 2.3. Biochemical Properties of Versiase

**Figure 3:**
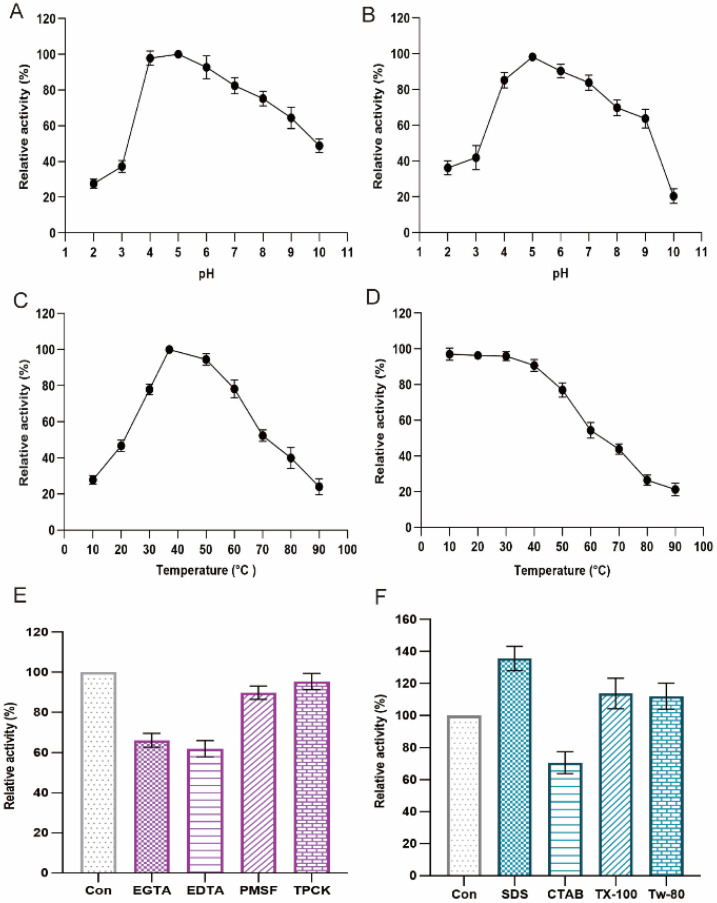
Biochemical properties of versiase. Optimum pH (**A**) and pH stability (**B**) were determined by assessing the enzyme activity in the pH range of 2.0~10.0 with various pH buffers. Temperature–activity relationship (**C**) and the thermostability (**D**) were determined with the range of 10~90 °C. Effects of protease inhibitors (**E**), and surfactants (**F**) were mixed with the corresponding inhibitors and surfactants. Versiase was mixed with the corresponding inhibitors (EGTA (5 mM), EDTA (5 mM), PMSF (5 mM), TPCK (10 mM), or surfactants (5 mM) at 40 °C, pH 5.0. All mixtures were incubated for 4 h. The relative activity was expressed as a percentage of the remaining activity to the control activity (without metal ions, inhibitors, or surfactants). Results are presented as mean ± SD (*n* = 3).

Effects of pH on the stability and activity of versiase were illustrated in Figure 3A,B, and the stability was determined at pH 2.0~10.0 by extending the conditions to 4 h. It was observed that versiase was more active in slightly acidic conditions with optimal pH around 5.0 (Figure 3A), which is different from the serine proteases that prefer neutral or alkali pH conditions [15,16,17]. It was found that versiase was relatively stable in a broad pH range over 4.0~8.0, with 85% of fibrinolytic activity maintained on average (Figure 3B).

The effects of temperature on versiase were shown in Figure 3C,D, and thermal stability was determined by incubation at 10~80 °C for 4 h. The maximum enzymatic activity was observed at 40 °C (Figure 3C), which is identical to that of the reported fibrinolytic enzymes [11,18,19]. The enzyme showed decent stability and retained nearly 80% of its original activity below 50 °C.

The activity of versiase was proven to be affected by corresponding inhibitors and surfactants. As shown in Figure 3E, the enzymatic activity of versiase was inhibited by metal chelating agents EDTA, EGTA, which is different from serine proteases that are sensitive to PMSF, TPCK [20]. Therefore, the fibrinolytic enzyme from *A. versicolor* ZLH-1 could be a metalloprotease.

A panel of surfactants, including SDS, Triton X-100, Tween 80, and CTAB, was also applied to challenge the fibrinolytic activity of versiase. Triton X-100, Tween 80 almost had no effect on the activity of versiase (Figure 3F). It was found that CTAB and SDS affect the enzymatic activity significantly; however, in two opposite ways: addition of SDS elevated the activity of versiase by 35.7%, whereas addition of CTAB lowered the enzymatic activity by 30%. This contrary effectiveness of the two surfactants may be due to their anionic or cationic natures that affect the binding of metals to versiase, given its accounted metalloprotease nature.

### 2.4. Fibrin(ogen)olytic Activity

**Figure 4:**
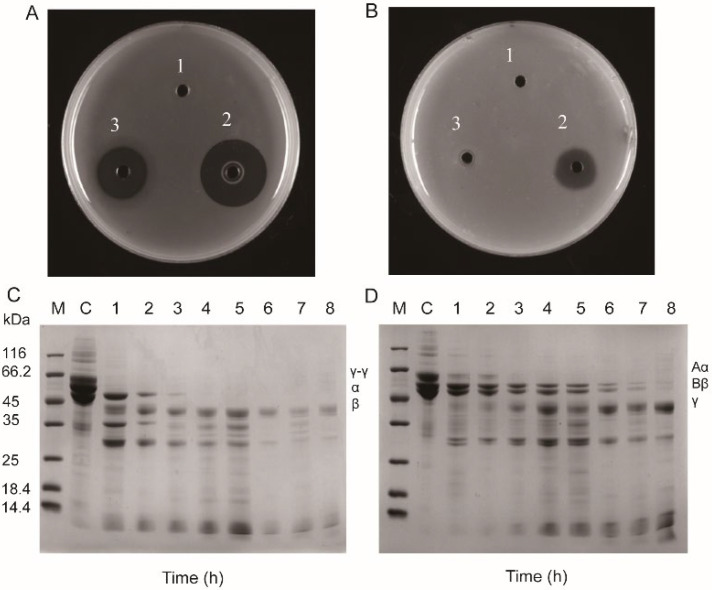
Fibrino(geno)lytic activity of versiase. Samples were applied to the wells in the plasminogen-rich fibrin plate (**A**) and plasminogen-free fibrin plate (**B**), 1. NB; 2. versiase (10.0 μg); 3. uPA (1000 U), plates then incubated at 37 °C for 4 h. (**C**) Degradation pattern of fibrin by versiase as analyzed by SDS-PAGE. The cross-linked fibrin was initiated by addition 200 μL of 1% human fibrinogen with 20 μL human thrombin (20 U/mL) dissolved in Tris-buffer (20 mM Tris-HCl (pH 7.2)). The fibrin clot was then hydrolyzed with 100 μL (13.82 μg/mL) of versiase. After incubation for indicated times, reaction mixtures were analyzed by 12% SDS-PAGE. (**D**) Cleavage pattern of human fibrinogen by versiase. One percent human fibrinogen was incubated with versiase and the conditions were consistent with (**C**). Aliquots were taken for 12% SDS-PAGE at different time intervals. Lanes: M—protein marker; C—Fibrinogen and the cross-linked fibrin incubated without versiase; 1–8, degradation pattern of fibrin(ogen) clot at different time intervals of 15, 30, 60, 100, 140, 180, 220, and 260 min.

The manner of versiase on fibrin hydrolyzing was investigated using both plasminogen-free and plasminogen-rich fibrin plates methods. The fibrin plate analysis was performed to qualitatively detect the degradation through the observation of the transparent area formed. As illustrated in Figure 4A,B, although both versiase and uPA (positive control) exhibited strong fibrinolytic activity (I.D.:22.864 mm and 17.735 mm) in plasminogen-rich fibrin plate, only versiase showed hydrolyzing activity (I.D.:10.607 mm) in the plate without plasminogen. Furthermore, transparent areas formed by versiase on the rich plate were bigger than that on the free plate. Taken together, the experimental results indicated that versiase exerted a bi-functional manner in hydrolyzing fibrin: it functioned directly as plasmin-like fibrinolytic enzyme or functioned indirectly as plasminogen activator. These findings are in line with the conclusions of bi-functional fibrinolytic enzymes activities, as documented in the reports on lumbrokinase [21], eupolytin 1 [22], and codiase [23].

Similar to other fibrinolytic enzymes, versiase could also hydrolyze all of the three chains of fibrin (ogen) [12,24]. As shown in Figure 4C, the γ-γ chains and α-chain of fibrin were cleaved rapidly by versiase, whereas the β-chain of fibrin was hydrolyzed slowly. Similarly, all the Aα, Bβ, and γ-chains of fibrinogen were susceptible to cleavage by versiase; Aα-chain was the fastest, followed by Bβ-chain and γ-chain (Figure 4D). It was observed that all hydrolyzed peptides derived from fibrin(ogen) by versiase were further degraded into smaller pieces. This sequential/or continuous hydrolyzation of versiase might reduce the side-effects, such as re-occlusion in clinic usage [25], given if approved for use.

### 2.5. Anticoagulant and Thrombolytic Activities

#### 2.5.1. Anticoagulant Activity In Vitro and In Vivo

**Figure 5:**
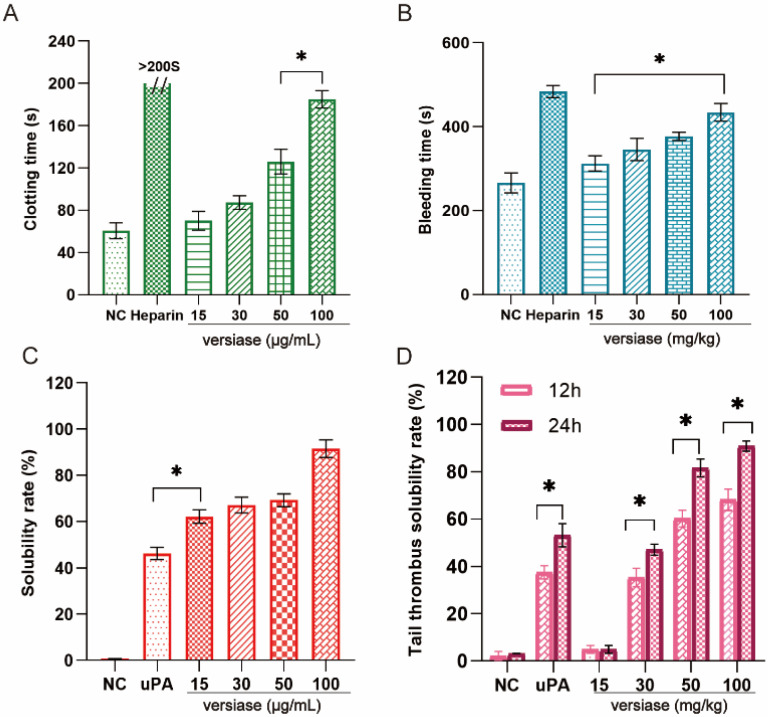
Anticoagulant and thrombolytic effects of versiase. (**A**) The anticoagulant effects of versiase in vitro; (**B**) the bleeding time after injected versiase; (**C**) the blood clots were hydrolyzed by versiase; (**D**) thrombolytic effect of versiase on tail thrombus model caused by carrageenan. The heparin were 10 μg/mL (**A**) and 1 mg/kg (**B**), uPA was 10,000 U (**C**,**D**). Results are presented as mean ± SD (*n* = 3). * *p* < 0.05.

Anticoagulant activity in vitro of versiase was assessed by measuring the clotting time on mice blood. The results indicated that no notable anticoagulant effect was observed at the concentration of 15 μg/mL, but the activity steadily enhanced as the concentration elevated (Figure 5A). The clotting time exceeded 185 s at 100 μg/mL.

In vivo anticoagulant assay, a positive correlation between the time and the dose of versiase was observed (Figure 5B). Addition of versiase extended the bleeding time in a dose-dependent manner, and the latter was extended significantly when treated with the high-end dose at 100 mg/kg of versiase (*p <* 0.05). The data suggested that versiase possessed a strong anticoagulant activity in vivo. It was noteworthy that the enzyme can also degraded circulating fibrinogen (Figure 4D), and this potential may lead to abnormal hemorrhage and the risk of bleeding may arise. Therefore, in reality, choices of dosage should be taken into consideration to prevent bleeding tendency.

#### 2.5.2. Thrombolytic Activity In Vitro and In Vivo

The thrombolytic activity of versiase was evaluated by observing the blood clots solubilization after the addition of versiase at varied doses in vitro (Figure 5C). The results indicated that applied doses of versiase lysed the clots effectively with a solubility percentage over 62%, even at low concentration at 15 μg/mL, higher than that of uPA (at 10,000 U) of 46% (*p* < 0.05). The solubility percentage went up in a dose-dependent manner, and it reached about 90% when the versiase dose was approaching to 100 μg/mL.

The tail thrombotic model was applied to test the in vivo thrombolytic ability of versiase. In experiments, thrombus formation was indicated by the color change of the tails of mice injected with carrageenan, and the appearance of auburn color marked the occurrence of thrombus. As shown in Figure 5D, no change in the thrombus length was noticed in the negative control group. However, the length of infracted regions of the thrombus was shortened significantly when the tail of mice was administered with versiase of 30 mg/kg and above by injection. Notably, the induced thrombus was completely scavenged after 24 h at the treatments with administration of higher doses of versiase at 50 and 100 mg/kg (Figure 5D and Figure S2). Interestingly, the antithrombosis effect of versiase at 50 mg/kg and above appeared to be better than that of uPA at 10,000 U.

In vitro and in vivo anticoagulant and thrombolytic experiments revealed that versiase could prolong the blood clotting time and hydrolyze the blood clot effectively in a concentration-dependent manner. The anticoagulant process is a highly interlinked array of multiple processes. It is either dependent on the inhibition of platelet aggregation or the intervention of the coagulation cascade. Our experiments demonstrate that versiase has anticoagulant properties; however, exactly which part of the process the enzyme works is unknown, and further work is still required. Although the molecular mechanism of antithrombotic effect is also unclear, it can be interpreted as its ability to degrade fibrinogen or fibrin, leading to a decrease in blood viscosity, resulting in prevention of thrombus formation or dissolving the already existing thrombus, possibly either due to activation of plasminogen, or degradation of the thrombus, directly or synergistically. These findings may reveal the potency of versiase as a worthy thrombolytic candidate for further investigation.

### 2.6. The Safety Evaluation

#### 2.6.1. The Hemolysis Test

**Figure 6:**
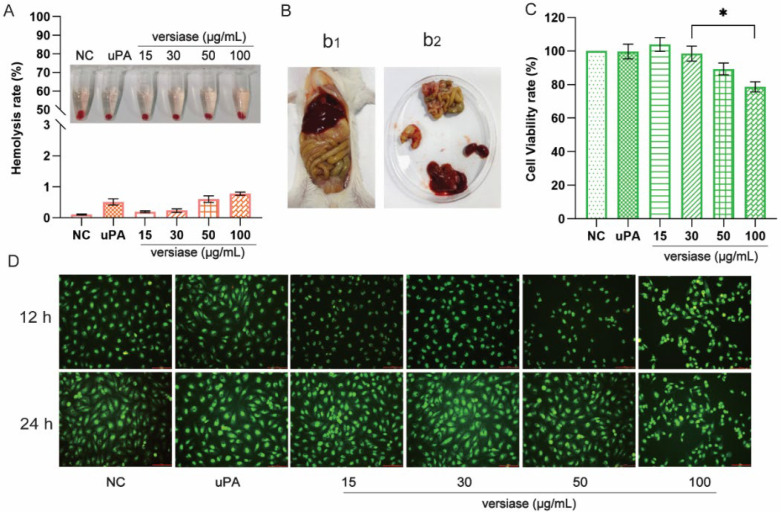
The safety evaluation of versiase. (**A**) The hemolysis of versiase; (**B**) the acute toxicity test. b1, abdominal cavity; b2, organs, three times intraperitoneal injection of versiase (400 μg) at the interval of 24 h; (**C**) the cytotoxicity of versiase on HUVECs; (**D**) live/dead fluorescent images (green: live cells, red: dead cells) of HUVECs after culturing with versiase for 12 and 24 h, and the scale bar is 100 μm. The uPA was 10,000 U. Results are presented as mean ± SD ((**A**–**C**) *n* = 3, D *n* = 6), * *p* < 0.05.

Hemolysis degree refers to the amount of hemoglobin released into plasma due to red blood-cell damage, and it is a common measure used for safety assessment. In experiments, varied doses of versiase were added to erythrocytes solution to check the hemolysis rate. The results demonstrated that no obvious hemolysis (normally >5%) took place with the additions of versiase at the tested levels (Figure 6A) [26]. In addition, the tested red blood cells remained intact and in normal shapes when being challenged with versiase at all the levels tested (Figure S3). Taken together, it could be concluded that versiase has very low tendency to cause hemolysis under given conditions, and thus it may meet with the low safety-concern needs once proven for further exploration.

#### 2.6.2. The Acute Toxicity In Vivo

To evaluate the acute toxicity of versiase in vivo, a high-concentration injection test was applied to observe the status of mice viscera. The results showed that no abnormal behavior observed for all the mice tested, and no obvious bleeding sighted in the abdominal cavity and major organs of the mice receiving versiase injections (Figure 6B). Taken together, it seems likely that versiase presents no obvious acute toxicity to mice in vivo.

#### 2.6.3. The Cytotoxicity Assay

The direct contact of fibrinolytic enzyme to vascular endotheliocyte usually rouses great concern, considering its potential cytotoxicity or other possible damages to the vascular walls. In the experiment, HUVECs was applied to evaluate the cytotoxicity of versiase in vitro (Figure 6C). The results showed that the cells challenged with versiase maintained a viability rate above 75% at all of the tested doses, which indicated that the use of versiase led to no cytotoxicity at the conditions applied.

The live/dead cell assay was also conducted to test the cytotoxicity of versiase. As shown in Figure 6D, a slight drop in the cell counting of HUVECs was observed with versiase applied at 50 and 100 μg/mL in the first 12 h. However, the number of cells bounced up when the cultivation was extended to 24 h. This turnover in cell counting suggests that versiase perhaps has some effect on cell division or proliferation, but merely in a short time-window, and once the enzymatic fibrinolytic ability of versiase vanished gradually over time, a resumption of cell normal proliferation occurs, as evidenced by the eventual increase in numbers of HUVECs cells. Actually, the dead cells (red) were barely invisible on the horizon compared to live cells (green) in all groups during the entire process. In conclusion, versiase was found to be safe and has no apparent cytotoxicity on HUVECs.

## 3. Materials and Methods

### 3.1. Microorganism and Cultivation

The fungal strain ZLH-1 was isolated from a marine sponge *Callyspongia* sp. collected in the sea area of Xuwen county, Guangdong province in the South China Sea, and was identified as *Aspergillus versicolor* and deposited at China General Microbiological Culture Collection Center (CGMCC) with accession number 23230.

*A. versicolor* ZLH-1 was stored on PDA slants at 4 °C, and before use, it was subcultured onto PDA plates and cultivated at 28 °C for 5 days. The liquid seed was prepared by adding a piece of the subculture agar into a 250 mL Erlenmeyer flask containing 50 milliliters of seed medium (potato 200 g, glucose 20 g, wheat bran 2.5 g, distilled water 1000 mL), and then it was incubated at 28 °C in a rotating shaker at 180 rpm for 16 h. The submerged production of enzymes was conducted by inoculating 2 mL of the liquid seed into the 250 mL Erlenmeyer flasks containing 50 mL production medium (potato 200 g, glucose 20 g, NaCl 15 g, distilled water 1000 mL), and the flasks were incubated in a rotating shaker at 28 °C and 180 rpm for 5 days.

### 3.2. Cells and Animals

The human Umbilical Vein Endothelial Cells line (HUVECs) was obtained from ATCC (Rockville, MD, USA), ATCC^®^ Number: CRL-1730. The cells were cultured in Ham’s F-12K medium (GIBCO, Gaithersburg, MD, USA). The media was supplemented with 10% FBS at 37 °C in 5% CO_2_ and changed every 2 days.

Male ICR mice (20–30 g, Pengyue laboratory animal Co., Ltd., Jinan, China) approximately 6~8 weeks old were used in all experiments. Food (Pengyue laboratory animal Co., Ltd., Jinan, China) and tap water were available ad libitum. All animal experimental protocols were approved (Approval No., OUC-SMP-20210404) and conducted by the Ethical Committee of Experimental Animal Care, Ocean University of China.

### 3.3. Preparation of the Fibrinolytic Enzyme

The fermentation medium was centrifuged at 6000 rpm for 20 min at 4 °C. Ammonium sulfate (AS) with various degrees of saturation (20~90%) was added to the supernatant, then the solution was kept at 4 °C for 12 h. The precipitation was collected by centrifugation at 10,000 rpm for 30 min at 4 °C; both the derived supernatant and precipitation were subjected to fibrinolytic enzyme activity testing. The relative enzymatic activity was calculated through the division of the measured activity of the precipitate or supernatant by the enzymatic activity of the original supernatant. AS was desalted using an ultrafiltration tube (1000 MWCO PSE, Sartorius, Göttingen, Germany).

The desalted protein was applied onto Uni^®^ Gel DEAE-80S (phenyl) column (1.0 × 10 cm), coupled with AKTA purifier UPC 100 system (GE Healthcare, Waukesha, WI, USA) balanced with 20 mM Tris-HCl buffer (pH 7.5). The bound proteins were eluted with linear gradient NaCl (0~1.0 M) at a flow rate of 1 mL/min. The fractions eluted were monitored by UV absorbance at 280 nm and assayed for fibrinolytic enzyme activity. The active fractions were collected, lyophilized, and used for further experiments. The purity of the enzyme was checked by sodium dodecyl sulfate polyacrylamide gel electrophoresis (SDS-PAGE). The purified enzyme was entitled as versiase hereafter.

The protein content was measured by a BCA protein assay [27] and serum albumin was used as a control, according to the manufacturer’s procedure (Sangon Biotech Co., Ltd., Shanghai, China).

### 3.4. Fibrinolytic Enzyme Activity Assay

The measurement of enzyme activity was conducted using the methods described by Krishnamurthy et al. with modification [28]. Briefly, the fibrin solution (1%) of 200 μL was mixed with 50 μL versiase solution and incubated at 37 °C for 1 h. After that, 400 μL of 0.4 M trichloroacetic acid solution was added and incubated at room temperature for 15 min to stop the reaction. The contents were centrifuged at 10,000 rpm for 15 min at 4 °C. The supernatant of 50 μL was mixed with 50 μL Folin phenol and 250 μL Na_2_CO_3_ solution, then the mixture was incubated at 40 °C for 20 min. The absorbance of the mix solution was measured at 680 nm by a microplate reader (BioTek, Winooski, VT, USA). A standard graph was made using gradient concentrations of tyrosine (0~100 μg/mL) with the same method as aforementioned. A unit (U) of fibrinolytic enzyme activity was defined as 1.0 μg of tyrosine liberated per min per ml under the standard assay conditions.

### 3.5. SDS-PAGE and Fibrin Zymography

The apparent molecular weight of the purified enzyme was determined by SDS-PAGE, of 5% stacking gel and 12% resolving gel [29], using a standard marker (Beyotime Biotech, Inc., Shanghai, China). The protein bands were observed by staining the gel with 1% Coomassie Brilliant Blue (CBB) R250 for 4 h and discolored immediately (water: acetic acid: ethanol = 17:2:1).

The versiase was also analyzed by fibrin zymography according to the method of Kim et al. [30]. The resolving gel solution (12%) was prepared with 0.12% (*w*/*v*) fibrinogen and thrombin (2 U/mL), whereas the purified enzyme sample was mixed with native gel sample loading buffer (5×). The electrophoresis was carried out in a cold water bath (~4 °C) at a constant voltage at 90 V. After electrophoresis, the gel was washed with 2.5% Triton X-100 solution at 4 °C for 1 h, followed by washing three times with PBS (0.01 M, pH 7.4) to remove Triton X-100. After, incubated at 37 °C for 12 h, the gel was stained with CBB and distained.

### 3.6. Protein Identification and Structure Prediction

Samples were obtained from clearly visible SDS-PAGE protein bands and analyzed by a proteomic approach using liquid-chromatography tandem mass spectrometry (LC-ESI-MS/MS). Firstly, the gel strip was cut and incubated with 40 μL trypsin buffer at 37 °C for 16~18 h. Then, the reaction solution was concentrated and desalted by using a C18 cartridges (Empore SPE, I.D.7 mm, volume 3 mL, Sigma). Further separation was conducted using a C18 reversed-phase column (Thermo Scientific Easy Column, 10 cm × 75 μm) of an UltiMate nano HPLC system, using a 60 min linear acetonitrile gradient (0.1% Formic acid). The column outlet was directly coupled to the ESI+ ion source of Q Exactive (Thermo Fisher, Waltham, MA, USA) mass spectrometer using a data-dependent top 10 method, dynamically choosing the most abundant precursor ions from the survey scan (300~1800 *m*/*z*) for HCD fragmentation. The normalized collision energy was 30 eV. The spectral data were analyzed by MaxQuant 1.5.51 (Max Planck Institute of Biochemistry, Martinsried, Germany; https://www.maxquant.org/) accessed on 23 June 2021, search against the NCBInr protein database (NCBI, Bethesda, MD, USA) accessed on 4 July 2021.

The prediction of protein secondary structure was made using tools in PredictProtein (http://www.predictprotein.org/) accessed on 7 July 2021, whereas the homology model was prepared by applying the online Swiss-model program (http://swissmodel.expasy.org/) [31] accessed on 8 July 2021.

### 3.7. Biochemical Characterization

The optimum pH and temperature of versiase were measured at the pH 2.0~10.0 and temperature of 10~80 °C, respectively. The pH stability and thermal stability of the purified enzyme were determined based on the residual fibrinolytic activity of the enzyme after incubation at pH 2.0~10.0 and 10~80 °C for 4 h.

The effect of different external factors on enzyme activity was evaluated, such as (1) inhibitors: 5.0 mM phenylmethylsulphonyl fluoride (PMSF), Ethylenebis(oxyethylenenitrilo)tetraacetic acid (EGTA), ethylenediaminetetraacetic acid (EDTA), 10.0 mM tosylphenylalnine chloromethyl keptone (TPCK); (2) surfactants: Triton X-100, Tween 80, sodium dodecyl sulfate (SDS), and Hexadecyl trimethyl ammonium Bromide (CTAB) was tested at 5.0 mM, respectively. Thereafter, the mixtures were incubated at 40 °C, pH 5.0 for 4 h.

### 3.8. Detection of Fibrinolytic Activity Using Fibrin Plate

Fibrinolytic activity was determined by the modified fibrin plate method [32] with slight modification, by using both plasminogen-free and plasminogen-rich plates. To prepare plasminogen-free fibrin plates, 300 mg of low-melting agar was dissolved in 15 mL Tris buffer (20 mM, pH 7.2), then the broth was added with 5 mL 25 mM CaCl_2_ solution, and slightly mixed with 10 mL fibrin solution (1%) and 20 μL thrombin (20 U/mL) at 50 °C. The mixture was poured into a sterile plate and spread carefully to obtain a homogeneous gel, then it was incubated at 70 °C for 1 h. Plasminogen-rich fibrin plate was prepared with the addition of an extra 5 units of plasminogen without heating [33]. To complete the fibrinolysis, 10.0 μg of versiase, and uPA (1000 U) were added into the wells (I.D. 3 mm) cut in the solidified agar plate, and then incubated at 37 °C for 4 h. Saline solution (0.9% NaCl) was used as a control. Finally, the transparent diameter was measured by using Image J v 1.8.0 software (USA National Institutes of Health).

### 3.9. Fibrin(ogen)olytic Assay

Fibrin(ogen)olytic activity of versiase was performed by following the method reported [23] with slight adjustments. Briefly, 200 μL of 1% fibrin (ogen) was incubated with 100 μL (13.82 μg/mL) of versiase in normal saline at 37 °C for varied times. The reaction was stopped by addition of denaturing buffer at the specified time. The digested products were analyzed by 12% SDS-PAGE.

### 3.10. Anticoagulant Effect of Versiase on Animal Blood In Vitro

Fresh blood (0.5 mL) of male ICR mice was collected, and varied doses of versiase were added with the same volume to get a series of final concentrations at 15, 30, 50, and 100 μg/mL, respectively. The negative and positive control was added with the same volume of normal saline and heparin (10 μg/mL), respectively. After gently mixing, the solutions were incubated at 37 °C to observe the formation of blood clot in the time span.

### 3.11. Examination of Anticoagulant Activity In Vivo

For anticoagulation evaluation, the tail transection test was performed by following the modified method reported [34]. Briefly, varied amounts of versiase (15, 30, 50, and 100 mg/kg) and heparin (1 mg/kg) were injected intravenously in the tail veins of mice; saline solution (0.9% NaCl) was used as a control. After 1 h, tail tips were cut at 3 mm and the tail was immediately immersed in distilled water at 37 °C. The bleeding time was determined by a visual endpoint method, in which the endpoint of bleeding was determined upon the time point that no redness appeared on the filter paper lightly touched by the tail dock.

### 3.12. In Vitro Thrombolytic Activities of Versiase

The clot lytic effect of versiase was investigated by using the blood clot test documented by Jun Yuan [35]. First, the male mice blood was allowed to coagulate naturally (25 °C) in a glass tube. Then, the derived clot was gently washed three times with saline solution and then weighed. An aliquot of 1 mL of varied doses of versiase (15, 30, 50, and 100 μg/mL), uPA (10,000 U) was added into the tubes with clot, respectively. The mixtures were incubated at 37 °C for 4 h. Then, the supernatant was removed and weighed. The blood clots remained in the tube. Saline solution (0.9% NaCl) was used as a control. The blood clot dissolution was determined by the formula as follows:

Solubility rate (%) = (W_i_ − W_r_)/W_i_ × 100% (1)

W_r_ was the weight of the remaining blood clot; W_i_ was the weight of the initial blood clot.

### 3.13. In Vivo Thrombolytic Test Using the Mouse Tail Thrombosis Model

Thrombolytic activity of versiase was examined using a carrageenan-induced mouse tail thrombosis model according to the methods reported [36]. The sterile carrageenan (10 mg/kg body weight) was dissolved in normal saline, and it was injected into the dorsal tail vein of male mice, ligated at 7 cm from the tip. The tail was kept at a low temperature (~15 °C) for 10 min and then the ligature was removed. The wine-red color formed at the tail tip indicated the formation of thrombus.

In experiments, the mice were randomly divided into 6 groups (*n* = 5). One hour after thrombus administration of carrageenan-induced, each experimental group was given versiase at set concentrations (15, 30, 50, and 100 mg/kg) by injecting the dorsal tail vein. The saline solution (0.9% NaCl) and uPA (10,000 U) were used as the control group. The thrombus length was measured at 0, 12, and 24 h after administration. The tail thrombus solubility was determined by the formula as follows:

Tail thrombus solubility rate (%) = (L_0_ − L_12 or 24_)/L_0_ × 100% (2)

L_0_ was the length of the tail thrombus at 0 h; L_12or 24_ was the length of the tail thrombus at 12 or 24 h.

### 3.14. The Hemolysis Test

Red blood cell suspension (2%) was prepared according to the method included in Chinese Pharmacopoeia (2010) [26], except for mouse blood applied instead of rabbit blood. The varied doses of versiase (30, 60, 100, and 200 μg/mL) and uPA (10,000 U) were added into the red blood cell suspensions by equal volume, mixed gently, and then the mixtures were incubated at 37 °C for 6 h. The same volumes of normal saline and 0.2% Triton X-100 were used as the negative and positive control, respectively. Afterwards, the absorbance of the supernatant was determined at 545 nm by the microplate reader. The morphology of erythrocytes was observed by light microscopy. The hemolysis effect was determined by the formula as follows:

Hemolysis rate (%) = (OD_t_ − OD_nc_)/(OD_pc_ − OD_nc_) × 100% (3)

OD_t_ was testing absorbances, OD_nc_ was negative absorbance, and OD_pc_ was positive absorbance.

### 3.15. The Acute Toxicity In Vivo

For toxicity evaluation, the tested mice were challenged by three-time intraperitoneal injection of versiase (400 μg) at the interval of 24 h. Then, the mice were observed regularly for any behavioral changes every 12 h, until 72 h. Moreover, the treated mice were checked for abdominal cavity and hemorrhage of main organs.

### 3.16. Analysis of Cell Viability

The effect of versiase on the viability of HUVEC cells was assessed by the MTT (3-(4,5-dimethylthiazol-2-yl)-2,5-diphenyltetrazolium bromide) assay [37]. The cells were seeded in 96-well plates (5 × 103 cells/well) and incubated for 24 h before the addition of versiase at varied concentrations (15, 30, 50, and 100 μg/mL). The PBS (0.01 M, pH 7.4) and uPA (10,000 U) were used as the negative and positive control, respectively. The medium was removed after 24 h of incubation, and 100 μL MTT solution (0.5 mg/mL) was added to each well for further incubation for 4 h. After that, the medium was carefully removed and then 100 μL dimethyl sulphoxide (DMSO) was added to each well. The absorbance of each well was measured at 570 nm using a microplate reader. Results were expressed as the percentage to the control.

The cells were stained by acridine orange/ethidium bromide (AO/EB) fluorochrome after being co-incubated for 12 or 24 h [38]. Cell viability images were observed using an inverted fluorescence microscope (Olympus, Tokyo, Japan).

### 3.17. Statistical Analysis

SPSS statistics 23 (IBM Corporation, Armonk, NY, USA) was applied for statistical analysis. Data were presented as means ± SD. Statistical analysis was performed using one-way analysis of variance (ANOVA) with Post Hoc Tukey HSD. Values of *p* < 0.05 were considered statistically significant.

## 4. Conclusions

In this study, for the first time we report the discovery of a fibrinolytic enzyme produced by marine isolate *A. versicolor* ZLH-1. The enzyme, versiase (37.3 kDa), was isolated from the fermentate and purified to homogeneity. Versiase belongs to the metalloprotease (M35) family, with maximum activity at 40 °C and pH 5.0. The unique bi-functional fibrinolytic manner facilitates versiase with an effective yet rapid capacity for dissolving blood clots, as well as with the thrombolytic and anticoagulant potencies. Versiase is concluded as safe for use thanks to its low hemolysis rate and negligible effect on the proliferation of HUVECs. The potential of versiase in treating thrombosis would be further explored once its thrombolysis and anticoagulant mechanisms are unveiled in the future investigations.

## Figures and Tables

**Table 1:** Two-step isolation and purification of the fibrinolytic enzyme from *A. versicolor* ZLH-1 culture supernate. Data represent a typical experiment.

Purification Steps	Volume (mL)	Protein (mg)	Activity (U)	Specific Activity (U/mg)	Recovery (%)	Fold
Supernate	500	770	24,244.8	31.5	100.0	1.0
AS (90%)	30	104	19,164.9	184.3	79.0	5.9
DEAE-(phenyl)	0.44	1.16	3905.9	3367.2	20.4	18.3

## Data Availability

The data presented in this study are available on request from the corresponding author.

## Supplementary Materials

The following supporting information can be downloaded at: https://www.mdpi.com/article/10.3390/md20060356/s1, Material S1: Figure S1: SDS-PAGE results for purification of fibrinolytic enzyme (versiase) from *A. versicolor* ZLH-1; Figure S2: Tail thrombosis in mice photographed at 24 h; Figure S3: The erythrocyte morphology photographed; Table S1: Mass spectrometry results for protein bands cut from CBB-stained SDS-PAGE gel from versiase of *A. versicolor* ZLH-1 with peptide information.

## References

[1] WHO (2021). Cardiovascular Diseases (CVDs), Fact Sheets. https://www.who.int/news-room/fact-sheets/detail/cardiovascular-diseases-(cvds).

[2] Mosesson M.W. (1992). The roles of fibrinogen and fibrin in hemostasis and thrombosis. Semin. Hematol..

[3] Danesh J., Lewington S., Thompson S.G., Lowe G.D., Collins R., Kostis J.B., Wilson A.C., Folsom A.R., Wu K., Benderly M. (2005). Plasma fibrinogen level and the risk of major cardiovascular diseases and nonvascular mortality: An individual participant meta-analysis. JAMA.

[4] Schrder R., Neuhaus K.L., Leizorovicz A., Linderer T., Tebbe U. (1987). A prospective placebo-controlled double-blind multicenter trial of intravenous streptokinase in acute myocardial infarction (ISAM): Long-term mortality and morbidity. J. Am. Coll. Cardiol..

[5] Lincoff A.M., Topol E.J. (1993). Illusion of reperfusion. Does anyone achieve optimal reperfusion during acute myocardial infarction?. Circulation.

[6] Altaf F., Wu S., Kasim V. (2021). Role of fibrinolytic enzymes in anti-thrombosis therapy. Front. Mol. Biosci..

[7] Wiman B. (1995). Plasminogen activator inhibitor 1 (PAI-1) in plasma: Its role in thrombotic disease. Thromb. Haemost..

[8] Flemmig M., Melzig M.F. (2012). Serine-proteases as plasminogen activators in terms of fibrinolysis. J. Pharm. Pharmacol..

[9] Marder V.J., Novokhatny V. (2010). Direct fibrinolytic agents: Biochemical attributes, preclinical foundation and clinical potential. J. Thromb. Haemost..

[10] Kotb E. (2014). The biotechnological potential of fibrinolytic enzymes in the dissolution of endogenous blood thrombi. Biotechnol. Prog..

[11] Nascimento T.P., Sales A.E., Porto T.S., Costa R.M.P.B., Breydo L., Uversky V.N., Porto A.L.F., Converti A. (2017). Purification, biochemical, and structural characterization of a novel fibrinolytic enzyme from Mucor subtilissimus UCP 1262. Bioproc. Biosyst. Eng..

[12] Choi J.-H., Sapkota K., Park S.-E., Kim S., Kim S.-J. (2013). Thrombolytic, anticoagulant and antiplatelet activities of codiase, a bi-functional fibrinolytic enzyme from *Codium fragile*. Biochimie.

[13] Li G., Liu X., Cong S., Deng Y., Zheng X. (2021). A novel serine protease with anticoagulant and fibrinolytic activities from the fruiting bodies of mushroom *Agrocybe aegerita*. Int. J. Biol. Macromol..

[14] Bogdanović X., Palm G.J., Schwenteit J., Singh R.K., Gudmundsdóttir B.K., Hinrichs W. (2016). Structural evidence of intramolecular propeptide inhibition of the aspzincin metalloendopeptidase AsaP1. FEBS Lett..

[15] Naveena B., Gopinath K.P., Sakthiselvan P., Partha N. (2012). Enhanced production of thrombinase by *Streptomyces venezuelae*: Kinetic studies on growth and enzyme production of mutant strain. Bioresour. Technol..

[16] da Silva A.V., do Nascimento J.M., Rodrigues C.H., Nascimento D.C.S., Costa R.M.P.B., Marques D.d.A.V., Leite A.C.L., Figueiredo M.d.V.B., Pastrana L., Converti A. (2020). Partial purification of fibrinolytic and fibrinogenolytic protease from *Gliricidia sepium* seeds by aqueous two-phase system. Biocatal. Agric. Biotechnol..

[17] Al Farraj D.A., Kumar T.S.J., Vijayaraghavan P., Elshikh M.S., Alkufeidy R.M., Alkubaisi N.A., Alshammari M.K. (2020). Enhanced production, purification and biochemical characterization of therapeutic potential fibrinolytic enzyme from a new *Bacillus flexus* from marine environment. J. King Saud Univ. Sci..

[18] Kumar S.S., Haridas M., Abdulhameed S. (2020). A novel fibrinolytic enzyme from marine *Pseudomonas aeruginosa* KU1 and its rapid in vivo thrombolysis with little haemolysis. Int. J. Biol. Macromol..

[19] Lu M., Gao Z., Xing S., Long J., Li C., He L., Wang X. (2021). Purification, characterization, and chemical modification of *Bacillus velezensis* SN-14 fibrinolytic enzyme. Int. J. Biol. Macromol..

[20] Liu X., Kopparapu N.-k., Shi X., Deng Y., Zheng X., Wu J. (2015). Purification and biochemical characterization of a novel fibrinolytic enzyme from culture supernatant of *Cordyceps militaris*. J. Agric. Food Chem..

[21] Mihara H., Sumi H., Yoneta T., MizuMOTO H., Ikeda R., Seiki M., Maruyama M. (1991). A novel fibrinolytic enzyme extracted from the earthworm, *Lumbricus rubellus*. Jpn. J. Physiol..

[22] Yang H., Wang Y., Xiao Y., Wang Y., Wu J., Liu C., Ye H., Li F., Yu H., Lai R. (2011). A bi-functional anti-thrombosis protein containing both direct-acting fibrin (ogen) olytic and plasminogen-activating activities. PLoS ONE.

[23] Matsubara K., Hori K., Matsuura Y., Miyazawa K. (2000). Purification and characterization of a fibrinolytic enzyme and identification of fibrinogen clotting enzyme in a marine green alga, *Codium divaricatum*. Comp. Biochem. Physiol..

[24] Majumdar S., Dutta S., Das T., Chattopadhyay P., Mukherjee A.K. (2015). Antiplatelet and antithrombotic activity of a fibrin (ogen) olytic protease from *Bacillus cereus* strain FF01. Int. J. Biol. Macromol..

[25] Chesebro J., Badimon L., Fuster V. (1988). The ischemic risk syndrome following thrombolysis: The problem of arterial re-occlusion. Curr. Opin. Cardiol..

[26] (2010). Chinese Pharmacopoeia.

[27] Smith P.K., Krohn R.I., Hermanson G.T. (1985). Measurement of protein using bicinchoninic acid. Anal. Biochem..

[28] Krishnamurthy A., Belur P.D. (2018). A novel fibrinolytic serine metalloprotease from the marine *Serratia marcescens* subsp. sakuensis: Purification and characterization. Int. J. Biol. Macromol..

[29] Laemmli U.K. (1970). Cleavage of structural protein during the assembly of the head of bacterio phase T4. Nature.

[30] Kim S.-H., Choi N.-S., Lee W.-Y. (1998). Fibrin zymography: A direct analysis of fibrinolytic enzymes on gels. Anal. Biochem..

[31] Waterhouse A., Bertoni M., Bienert S., Studer G., Tauriello G., Gumienny R., Heer F.T., de Beer T.A.P., Rempfer C., Bordoli L. (2018). SWISS-MODEL: Homology modelling of protein structures and complexes. Nucleic Acids Res..

[32] Kim J.B., Jung W.H., Ryu J.M., Lee Y.J., Jung J.K., Jang H.W., Kim S.W. (2007). Identification of a fibrinolytic enzyme by *Bacillus vallismortis* and its potential as a bacteriolytic enzyme against *Streptococcus mutans*. Biotechnol. Lett..

[33] Lassen M. (1953). Heat Denaturation of Plasminogen in the Fibrin Plate Method. Acta Physiol..

[34] Tucker E.I., Verbout N.G., Leung P.Y., Hurst S., McCarty O.J., Gailani D., Gruber A. (2012). Inhibition of factor XI activation attenuates inflammation and coagulopathy while improving the survival of mouse polymicrobial sepsis. Blood JASN.

[35] Yuan J., Yang J., Zhuang Z., Yang Y., Lin L., Wang S. (2012). Thrombolytic effects of Douchi Fibrinolytic enzyme from *Bacillus subtilis* LD-8547 in vitro and in vivo. BMC Biotechnol..

[36] Bekemeier H., Hirschelmann R., Giessler A. (1984). Carrageenin-induced thrombosis in the rat and mouse as a test model of substances influencing thrombosis. Biomed. Biochim. Acta.

[37] Gerlier D., Thomasset N. (1986). Use of MTT colorimetric assay to measure cell activation. J. Immunol. Methods.

[38] Kasibhatla S., Amarante-Mendes G.P., Finucane D., Brunner T., Green D.R. (2006). Acridine Orange/Ethidium Bromide (AO/EB) Staining to Detect Apoptosis. CSH Protoc..

