# Supplementary material for: A Novel Bi-Functional Fibrinolytic Enzyme with Anticoagulant and Thrombolytic Activities from a Marine-Derived Fungus Aspergillus versicolor ZLH-1
Source: Mar Drugs. 2022 May 27;20(6):356. doi: 10.3390/md20060356 (PMC9229710; doi:10.3390/md20060356)
Supplement: Supplementary file 1 [file marinedrugs-20-00356-s001.zip › 2-Supplementary Material 1.pdf]

marine drugs

MDPI

Supplementary material for:

Characterization of a novel bi-functional fibrinolytic enzyme with anticoagulant and thrombolytic activities by marine fungus *Aspergillus versicolor* ZLH-1

By

Lihong Zhao[a], Xiuping Lin[b], Jingyun Fu[a], Jun Zhang[a], Wei Tang[a], Zengguo He[a,c,d *]

a School of Medicine and Pharmacy, Ocean University of China, Qingdao 266003, China.

b CAS Key Laboratory of Tropical Marine Bio-resources and Ecology, Guangdong Key Laboratory of Marine Materia Medica, South China Sea Institute of Oceanology, Chinese Academy of Sciences, Guangzhou, 510301, China.

c Marine Biomedical Research Institute of Qingdao, Qingdao 266071, China.

d Qingdao Bioantai Biotechnology Co.,Ltd. , Qingdao 266000, China.

[*]Corresponding author at: School of Medicine and Pharmacy, Ocean University of China, Qingdao 266003, China. Tel & Fax: +86-186-1113-7588; E-mail address: bioantai88@vip.163.com (Zengguo He).

1. Description of content

Figure S1. Crude extract precipitation with ammonium sulfate (AS) up to eight saturation ranges, from 20% to 90%. The resulted of protein in SDS-PAGE (Fig. S1A&B) showed that near all the proteins in fermentation supernatant were deposited at 90% AS saturation. Since fibrinolytic activity was mainly retained in the 90% saturation, so used 90% AS saturation to get the crude enzyme.

The result of DEAE-80S(phenyl) ion exchange column purified enzyme was detected by SDS-PAGE method, the results were detected every 2 minutes, and shown in Fig. S1C. Furthermore, SDS-PAGE (Fig. S2C) of the eluate from this step showed the presence of only one protein lane between lanes 34 and 46。

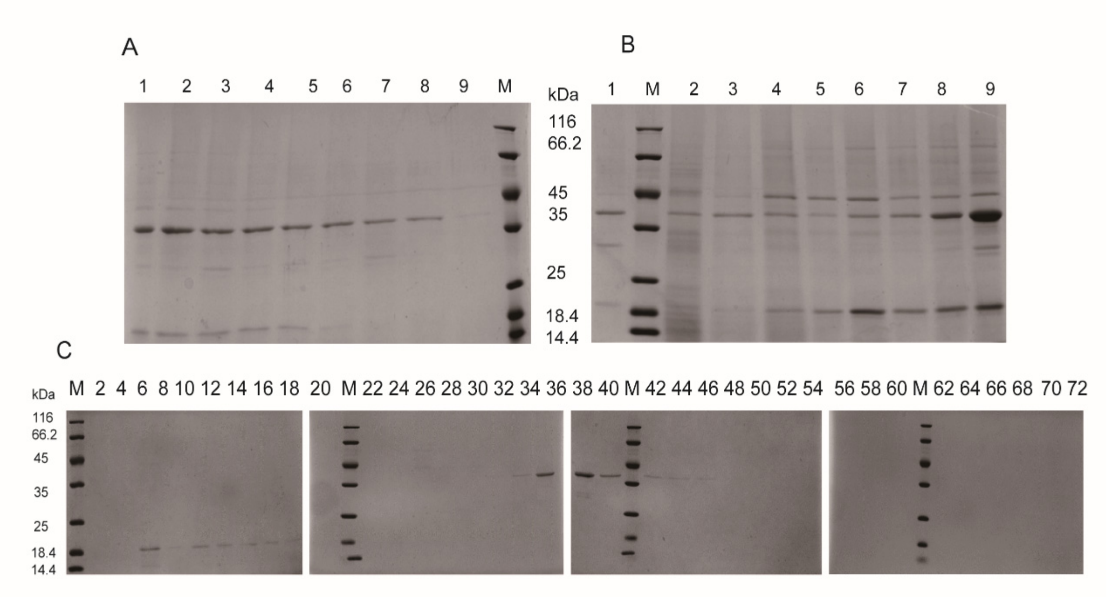

Figure S1. SDS-PAGE results for purification of fibrinolytic enzyme (versiase) from *A. versicolor* ZLH-1. A. SDS-PAGE results of the original supernatant and different saturation of AS supernatant. B. SDS-PAGE results of the original supernatant and different saturation of AS precipitation. A&B: Lanes: M, protein marker; 1-9 was corresponding ammonium sulfate saturation 0, 20, 30, 40, 50, 60, 70, 80, 90%, respectively. C. SDS-PAGE results for different times of DEAE-80S(phenyl) ion exchange column. Lanes: M, protein marker; 2-72 was corresponding time of Fig. 1B.

Figure S2. The carrageenan-induced tail thrombosis picture of mouse at 24 h post versiase administration.

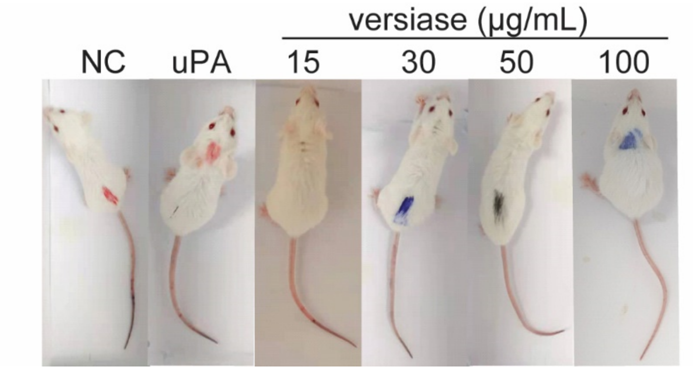

Figure S2. Tail thrombosis in mice photographed at 24 h. The NC (negative control) and uPA (positive control, 10000 U) were represent, and four concentrations of versiase (15, 30, 50, and 100 μg/mL) were studied.

Figure S3. Afterwards test the supernatant absorbance of hemolysis, then gently mix the rest of each tube, taking 20 μL mixture and observe it directly under the microscope (Olympus, Japan) (Fig.S3).

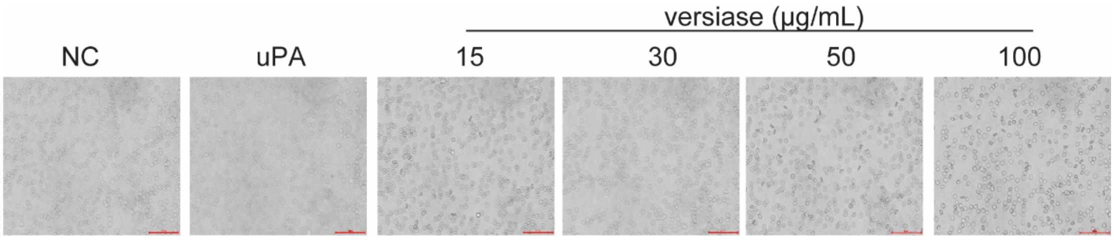

Figure S3. The erythrocyte morphology photographed. The NC (negative control) and uPA (positive control, 10000 U) were represent, and four concentrations of versiase (15, 30, 50, and 100 μg/mL) were studied. The scale bar is 100 μm.
